# Application of machine learning algorithms for multiparametric MRI-based evaluation of murine colitis

**DOI:** 10.1371/journal.pone.0206576

**Published:** 2018-10-26

**Authors:** Stephan Ellmann, Victoria Langer, Nathalie Britzen-Laurent, Kai Hildner, Carina Huber, Philipp Tripal, Lisa Seyler, Maximilian Waldner, Michael Uder, Michael Stürzl, Tobias Bäuerle

**Affiliations:** 1 Institute of Radiology, University Hospital Erlangen, Maximiliansplatz 1, Erlangen, Germany; 2 Division of Molecular and Experimental Surgery, Translational Research Center Erlangen, Department of Surgery, Erlangen, Germany; 3 Department of Medicine 1, University Hospital Erlangen, Kussmaul Campus for Medical Research, Erlangen, Germany; 4 Optical Imaging Center Erlangen (OICE), Friedrich-Alexander-University Erlangen-Nuremberg (FAU), Erlangen, Germany; University of Queensland, AUSTRALIA

## Abstract

Magnetic resonance imaging (MRI) allows non-invasive evaluation of inflammatory bowel disease (IBD) by assessing pathologically altered gut. Besides morphological changes, relaxation times and diffusion capacity of involved bowel segments can be obtained by MRI. The aim of this study was to assess the use of multiparametric MRI in the diagnosis of experimentally induced colitis in mice, and evaluate the diagnostic benefit of parameter combinations using machine learning. This study relied on colitis induction by Dextran Sodium Sulfate (DSS) and investigated the colon of mice *in vivo* as well as *ex vivo*. Receiver Operating Characteristics were used to calculate sensitivity, specificity, positive- and negative-predictive values (PPV and NPV) of these single values in detecting DSS-treatment as a reference condition. A Model Averaged Neural Network (avNNet) was trained on the multiparametric combination of the measured values, and its predictive capacity was compared to those of the single parameters using exact binomial tests. Within the *in vivo* subgroup (n = 19), the avNNet featured a sensitivity of 91.3% (95% CI: 86.6–96.0%), specificity of 92.3% (95% CI: 85.1–99.6%), PPV of 96.9% (94.0–99.9%) and NPV of 80.0% (95% CI: 69.9–90.1%), significantly outperforming all single parameters in at least 2 accuracy measures (p < 0.003) and performing significantly worse compared to none of the single values. Within the *ex vivo* subgroup (n = 30), the avNNet featured a sensitivity of 87.4% (95% CI: 82.6–92.2%), specificity of 82.9% (95% CI: 76.1–89.7%), PPV of 88.9% (84.3–93.5%) and NPV of 80.8% (95% CI: 73.8–87.9%), significantly outperforming all single parameters in at least 2 accuracy measures (p < 0.015), exceeded by none of the single parameters. In experimental mouse colitis, multiparametric MRI and the combination of several single measured values to an avNNet can significantly increase diagnostic accuracy compared to the single parameters alone. This pilot study will provide new avenues for the development of an MR-derived colitis score for optimized diagnosis and surveillance of inflammatory bowel disease.

## Introduction

Inflammatory bowel diseases (IBD)–mainly consisting of Crohn’s disease (CD) and ulcerative colitis (UC)–are persistent or recurrent intestinal inflammations affecting the entire gastrointestinal system or the colonic mucosa, respectively [1]. As a common pathomechanism, genetically susceptible hosts feature deregulated mucosal T cell responses to enteric bacteria [2]. The details of these genetic-environmental-immunological interactions are still not resolved. However, there is consent that CD is characterized by a rather Th1 immune response and submucosal T-cell-infiltration, whereas UC evokes a Th2-dominated immune response with mucosal infiltration [3,4]. However, typical Th1 cytokines like tumor necrosis factor alpha (TNF-α) and interferon gamma (IFN-γ) also arise in UC [5,6].

Experimental animal models are commonly used to investigate the pathogenesis of IBD. Specifically dextran sulphate sodium (DSS)-induced colitis is a commonly used model of murine colitis and many drugs used in IBD patients have been developed with the help of this model [7–10]. DSS-induced colitis in mice closely resembles the morphological and symptomatic features of human UC [11], with a predominant affection of the mucosa and the distal left colon, but often extending throughout the entire colon. As yet, the diagnosis of the disease—in humans and animals—is based on clinical characteristics as well as endoscopic and histologic mucosal features. Colonoscopy allows direct visualization of the colonic mucosa, but is invasive and can cause complications [12].

In recent years, imaging techniques are increasingly considered as a tool to improve diagnosis and surveillance of IBD patients. Whilst computed tomography (CT) is a widely available and fast method, it lacks sensitivity in terms of detection of early mucosal changes, and is associated with the risks of repeated radiation exposure. Due to the technical progress of the last years, magnetic resonance imaging (MRI) has become the imaging modality of choice in detection, surveillance, therapy monitoring and evaluation of the extent of IBDs in humans [13,14]. However, bowel imaging in animal models remains challenging.

Several studies reported on the perspectives of MRI in murine colitis imaging [15–21]. Of note, these studies mainly investigated single variables like wall thickness or relaxation times, but did not investigate the additional benefit of multiple variables, parameter combinations and assets of machine learning algorithms.

This study describes *in* and *ex vivo* MRI protocols for imaging of DSS-induced colitis in mice, and presents a multiparametric approach for colitis detection based on a machine learning algorithm. By this approach, increased diagnostic accuracy as compared to single parameter analyses was obtained.

## Materials and methods

### Animals

C57BL/6 mice were obtained from Charles River, Germany, and housed at the central animal facility of the University of Erlangen-Nuremberg. The animals were kept in standard laboratory cages in groups of three or four per cage. To avoid potential interfering infections, mice were kept isolated and were fed with pathogen-free food. Clinical symptoms including body weight, rectal bleeding, behavior, appearance and general health condition were monitored daily. All care and experimental procedures were performed in accordance with national and regional legislation on animal protection, and all animal procedures were approved by the State Government of Middle Franconia, Germany (reference numbers 54–2532.1-12/12 and 55.2–2532.1-37/14). For tissue histology and *ex vivo* imaging, mice were sacrificed by cervical dislocation under isoflurane anesthesia (2%, 2 L/min). In total, 43 mice were used.

### Colitis induction

Acute colitis was induced in sex-matched co-housed littermates with a minimal body weight of 20 g by administration of 2.5% DSS (36–50 kDa; MP Biomedicals) in the drinking water for a 7-day cycle, followed by 3 days of normal drinking water. Control animals received tap water only. Animals were randomly assigned to DSS- or sham-treatment. DSS-induced colitis was evaluated on day 9 by endoscopy while MRI was performed on day 10.

In addition, colon of control as well as DSS-treated animals were prepared from caecum to rectum and used for *ex vivo* MRI analysis and immunohistochemistry.

### Colitis evaluation using endoscopy and clinical examination

*In vivo* endoscopy was used to evaluate the DSS-induced colitis grade. Mice were anaesthetized with isoflurane (2%, 2 L/min) and a high-resolution mini endoscope including a xenon light source and an air pump (Karl-Storz, Germany) was used to visualize the intestinal mucosa at the level of the rectosigmoid junction by blinded investigators (VL and CH). Disease activity was evaluated according to Becker et al. [22] with a disease specific scoring system using translucency of the colon wall, granularity of the mucosal surface, fibrin deposition, vascularization and stool consistency as parameters. Each parameter was given a score from 0 to 3, summing up to a maximum score of 15 indicative of strong colitis.

### Histology

Colon specimen of 21 mice were fixed overnight in 4% paraformaldehyde, embedded in paraffin and cut into 4 μm sections. Hematoxylin/Eosin staining was performed to visualize tissue morphology and inflammation. Slides were assessed for lymphocytic cell infiltration (0–3 points) and tissue damage (0–3 points) at the level of the rectosigmoid junction by a blinded investigator (MW). The resulting points were summed up to receive a histology score ranging from 0–6.

### *In vivo* MRI

19 mice (13 mice with DSS-induced colitis, 6 controls) received Isoflurane anesthesia (2%, 2 L/min) and an intraperitoneal injection of butylscopolamine (Buscopan, Boehringer Ingelheim, Germany, 5 mg/kg body weight) prior to MRI examination. In addition, the distal colon was gently flushed with saline solution and subsequently filled with a carob gum/saline solution mixture (1%). Imaging was performed on a 7 Tesla Bruker ClinScan MRI with the sequences listed in Table 1 with a maximum gradient strength of 660 mT/m and a maximum slew rate 4570 T/m/s. The automatic shimming procedure of the Bruker ClinScan MRI was used to achieve sufficient shimming, offering room temperature resistive 1^st^ and 2^nd^ order shims [23].

**Table 1:** *In vivo* MRI sequences.

	T1	T2	T1 Map	T2 Map	T2* Map	ADC Map
Sequence	SE	SE	FLASH	SE	GRE	EPI
Orientation	axial	axial	axial	axial	axial	axial
TR (ms)	530	3890	50	3410	2000	5000
TE (ms)	9	56	2.5	10.2	4	31
Averages	2	1	1	1	1	4
FA (°)	90	140	8 / 42	180	40	90
FOV (mm)	24 × 35	24 × 35	25 × 35	25 × 35	25 × 35	23 × 34
Slices	40	35	36	35	40	35
Slice thickness (mm)	0.7	1.0	1.0	1.0	1.0	1.0
Matrix	512 × 360	320 × 224	320 × 230	192 × 136	384 × 276	100 × 68
Duration	05:34	01:18	08:22	07:42	09:12	04:25

Acquired sequences included T1w and T2 sequences, T1-, T2-, T2*- and ADC maps.

Abbreviations: Spin echo (SE), fast low angle shots (FLASH), gradient echo (GE), echoplanar imaging (EPI) for determination of the apparent diffusion coefficient (ADC). Repetition time (TR), time to echo (TE), flip angle (FA), field of view (FOV). Acquisition duration is given in min:sec.

### *Ex vivo* MRI

The colons of 30 sacrificed mice (18 mice with DSS-induced colitis, 12 controls) were prepared and embedded in agarose dissolved in saline solution (2%). *Ex vivo* imaging was performed on the same system as *in vivo* imaging with the sequences listed in Table 2.

**Table 2:** *Ex vivo* MRI sequences.

	T1	T2	T1 Map	T2 Map	T2* Map	ADC Map
Sequence	SE	SE	FLASH	SE	GRE	EPI
Orientation	axial	axial	axial	axial	axial	axial
TR (ms)	941	13300	50	7082	2500	8000
TE (ms)	14	43	2.5	10.2	4	60
Averages	15	4	2	1	2	1
FA (°)	180	180	8 / 42	180	40	90
FOV (mm)	28 × 28	28 × 28	28 × 28	28 × 28	28 × 28	28 × 28
Slices	60	80	40	40	38	40
Slice thickness (mm)	1.0	0.5	1.0	1.0	1.0	1.0
Matrix	512 × 512	512 × 512	512 × 512	512 × 512	512 × 512	100 × 100
Duration	174:15	131:27	41:18	45:27	42:40	16:16

Acquired sequences included T1w and T2 sequences, T1-, T2-, T2*- and ADC maps.

Abbreviations: Spin echo (SE), fast low angle shots (FLASH), gradient echo (GE), echoplanar imaging (EPI) for determination of the apparent diffusion coefficient (ADC). Repetition time (TR), time to echo (TE), flip angle (FA), field of view (FOV). Acquisition duration is given in min:sec.

### Image analysis

Images were analyzed with Horos [24]. For *in vivo* as well as *ex vivo* imaging, region of interest (ROI) measurements were performed in the wall of the distal colon (n = 10 per animal). For this purpose, ROIs were placed within the bowel walls in the T2w sequences by a blinded investigator (SE), carefully avoiding the lumen of the colon or surrounding fat tissue (or agarose in case of *ex vivo* analyses). All measurements were acquired around the rectosigmoid junction (approx. ±3 mm) where the colon was orientated perpendicular to slice orientation. The ROIs were copied to the other sequences (T1, T2, T2* and ADC Maps), and slightly adjusted if necessary (e.g. due to bowel movements altering the distinct location of the bowel segment to measure). Measurement of the colon wall thickness was performed in the T2w sequences with the distance tool.

### Statistical analysis and machine learning

Statistical analyses were performed using RStudio [25]. Normal distribution of data was assessed using Kolmogorov-Smirnov-tests. For the comparison of means between groups t-tests were applied for normally distributed data, and Mann-Whitney U tests to compare the medians of data that significantly differed from normal distribution. Linear correlations were calculated with the Pearson correlation method.

Machine learning model development and implementation was performed using the caret package for R [26]. DSS-treatment was used as reference condition with the aim to predict the dichotomous outcome (DSS-treated vs. sham-treated) from the set of predictors wall thickness, T1-, T2-, T2*-relaxation times, apparent diffusion coefficient (ADC) and the type of examination (*in vivo* vs. *ex vivo*).

To assess the model’s ability to predict unknown data, a modified Leave-one-out-cross-validation was applied: All measurements obtained from one animal were eliminated from the dataset and treated as a test-set, the model was trained with the remaining data (train-set) which was then used to predict the outcome of the formerly eliminated data. This was performed consecutively for all animals in a walk-through-fashion, so that in the end the complete dataset underwent prediction of the outcome by models trained with data not part of the test-set. Within this process, the partially correlated predictor parameters were subjected to a principal component analysis (PCA) to convert the set of observations into a set of values of linearly uncorrelated variables (S1 Fig). The resulting principal components were then fed into several machine learning algorithms to assess their diagnostic accuracy in discriminating between DSS-treated and sham-treated animals. Model Averaged Neural Networks (avNNet) were further evaluated due to their high accuracy in this screening procedure.

Neural networks are combinations of neurons organized in layers with the predictors as the bottom layer, and the output as the top layer. The applied avNNet features one additional intermediate layer containing hidden neurons as nodes, receiving input from the predictors and forming the output. The inputs to each node are combined using a weighted linear combination. The result is then modified by a nonlinear function before being returned as output. The values of the weights have to be restricted to prevent them from becoming too large, and the parameter restricting the weights is referred to as decay. The initial weights are chosen randomly and updated during the training process using the observed data. Consequently, there is a certain amount of randomness in all predictions [26]. To account for this, the network was trained 5 times using different random starting points, and the resulting data were averaged.

To assess the predictive abilities of every single parameter alone, Receiver-Operating Characteristic (ROC) analyses were performed for the single predictors, and optimal sensitivity and specificity values were calculated from the ROC curves by the Youden method.

The avNNet and the predictive abilities of all single parameters alone were compared to each other by exact binomial tests using the R package DTComPair version 1.0.3 [27].

In all statistical tests, p-values < .05 were considered statistically significant.

Finally, a model was trained on the complete dataset with a decay value of 0.015 and 7 hidden neurons. The training process was validated with a 10 times repeated 10-fold cross-validation. The resulting model was implemented into a publically accessible internet application using Shiny [28].

## Results

Mice with DSS-induced colitis and control animals were comparatively investigated using clinical evaluation methods, histology and MRI.

### DSS-induced colonic inflammation

**Fig 1:**
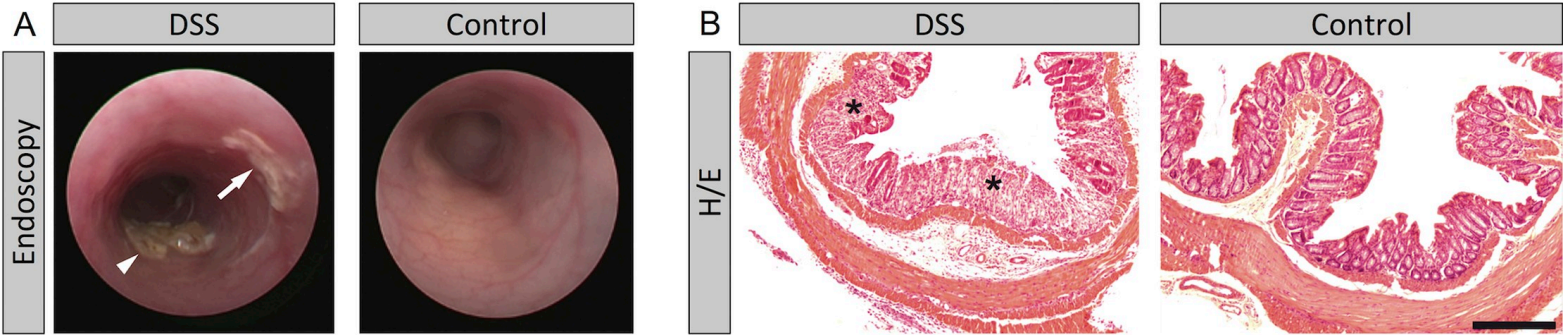
Comparison of colonoscopy and histology between DSS- and sham-treated animals. (A) Representative colonoscopy of a DSS-treated animal and a control animal. In contrast to controls, DSS-treated animals featured lower colonic wall translucency and vascularization, higher granularity, diarrhea (arrowhead) and fibrin deposition (arrow). (B) Representative colon histology images (H/E staining) of animals treated with DSS and control animals (scale bar 250 μm). Treatment with DSS induced a strong inflammation in the colon (asterisks).

**Fig 2:**
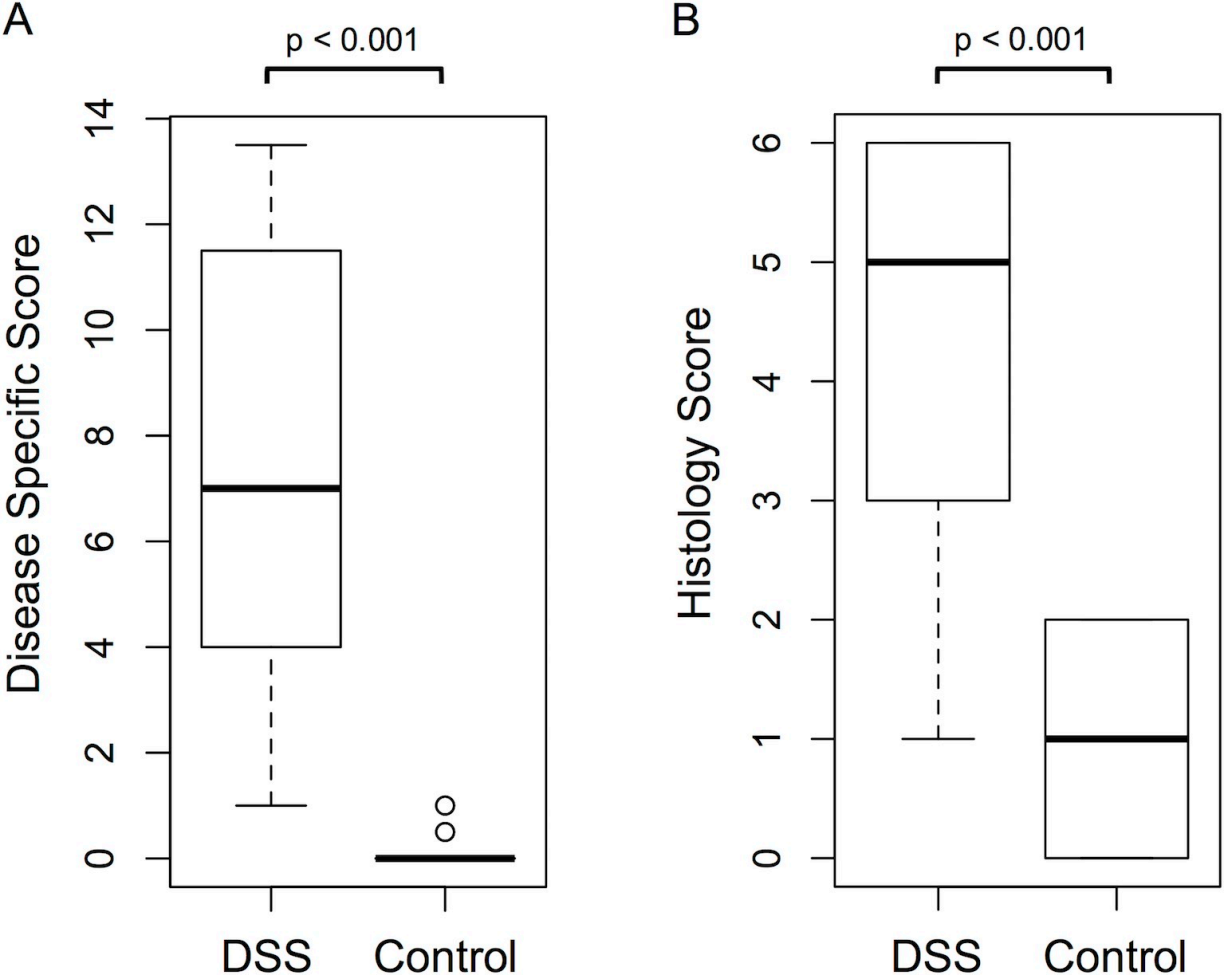
Boxplot comparison of disease specific scores and histology scores between DSS- and sham-treated animals. DSS-treated animals featured (A) significantly higher disease specific scores than control animals (median 7 vs. 0, p < 0.001; n = 43) and (B) higher histology scores (median 5 vs. 1, p < 0.01; n = 21). Boxplots follow the Tukey definition.

In DSS-treated animals as compared to control animals colonic wall translucency, granularity, fibrin deposition and vascularization were changed (Fig 1A). This was accompanied by diarrhea and significantly higher disease specific scores in DSS-treated animals (median 7 vs. 0, p < 0.001, Fig 2A) and higher histology scores (median 5 vs. 1, p < 0.001, Fig 2B). In accordance to endoscopic results, histological evaluation of the colon of DSS-treated mice showed crypt distortion and cell damage, an increased immune cell infiltration, and edema (Fig 1B).

**Fig 3:**
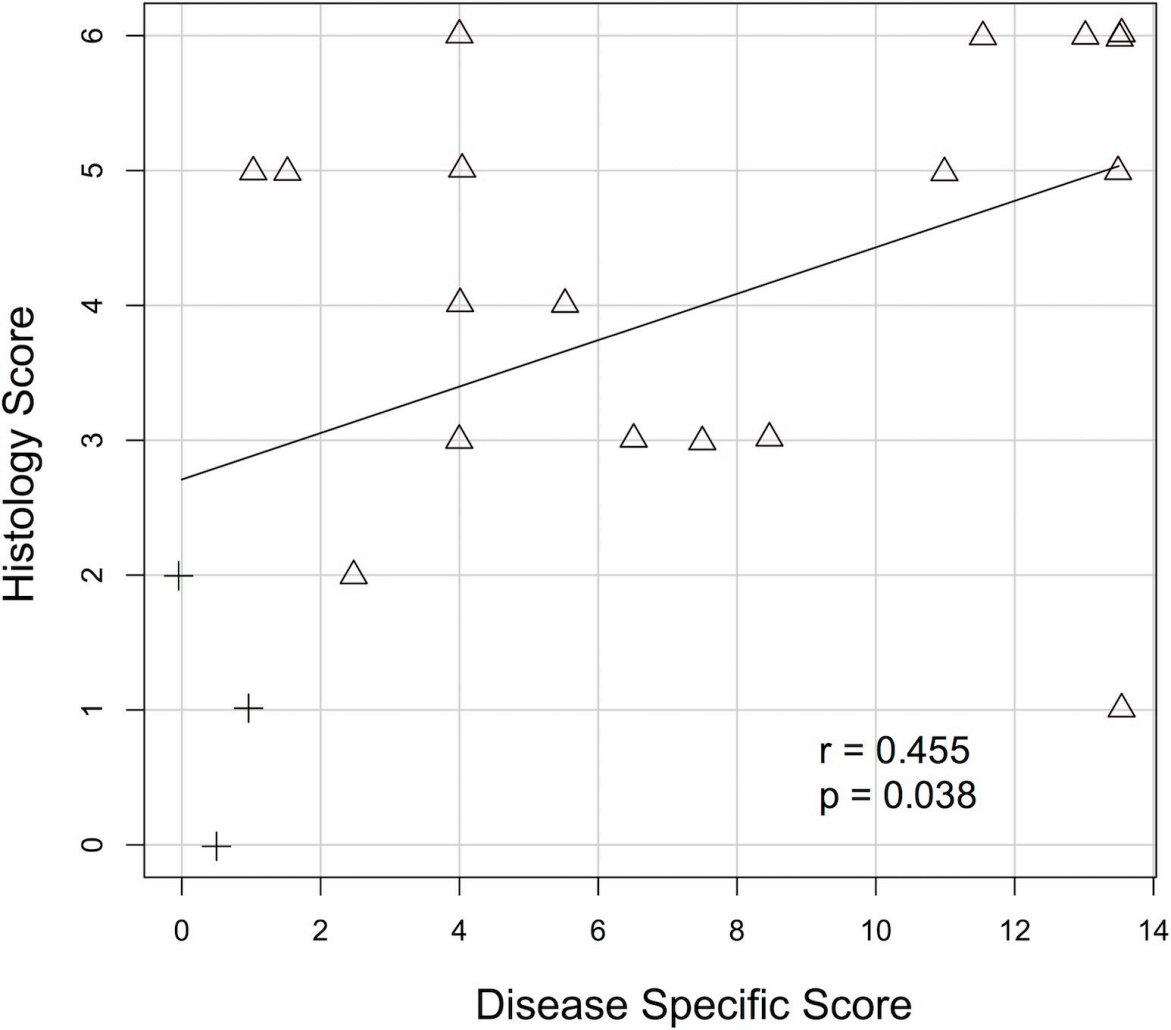
Pearson correlation plot of disease specific scores and histology scores. DSS-treated animals are depicted with triangles, sham-treated animals are depicted with crosses. A moderate but significant correlation was observed (r = 0.455, p = 0.038).

Disease specific scores correlated with histology scores moderately but significantly (Fig 3, r = 0.455, p = 0.038). Of notice, sham-treated animals featured histology scores of up to 2 points, animals with disease specific scores ≤4 exhibited histology scores ranging from 0–6, and one animal featured a disease specific score of 13.5 but a histology score of only 1.

### Subjective MRI analysis

**Fig 4:**
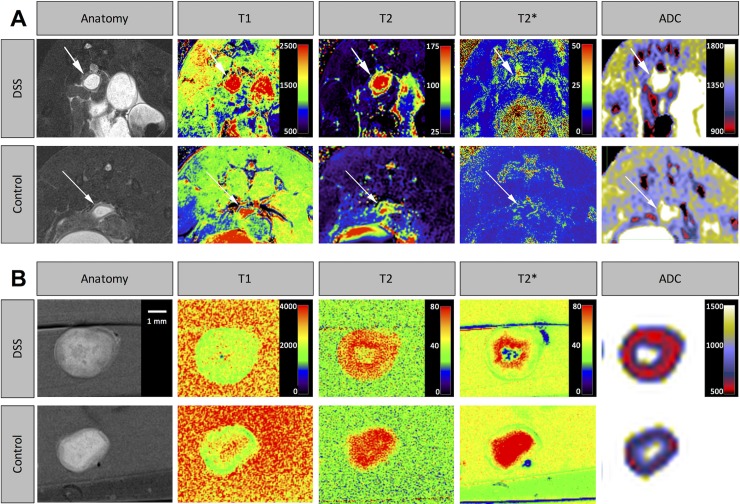
Representative magnetic resonance (MR) images. (A) *in vivo* MR images of the distal colon in a T2-TSE sequence, T1-, T2- and T2*-mapping and an ADC map for a DSS-treated and a control animal (upper and lower row, respectively). The colon wall is marked with an arrow. The walls of DSS-treated animals featured increased thickness, higher T1- and T2-relaxation times and higher ADC values (compare Table 3 and Fig 5). (B) *ex vivo* MR images of the distal colon in a T2-TSE sequence, T1-, T2- and T2*-mapping and an ADC map for a DSS-treated and a control animal (upper and lower row, respectively). The walls of DSS-treated animals featured increased thickness, increased T2-relaxation times and reduced ADC values (compare Table 3 and Fig 5).

In an initial analysis of the image material, distal colonic segments were identified that were adequately filled with carbon gum solution and free from motion artifacts in all sequences (Fig 4). Those segments were used for relaxation time and ADC measurements within the acquired maps and determination of the colon wall thickness.

### Multiparametric imaging

**Fig 5:**
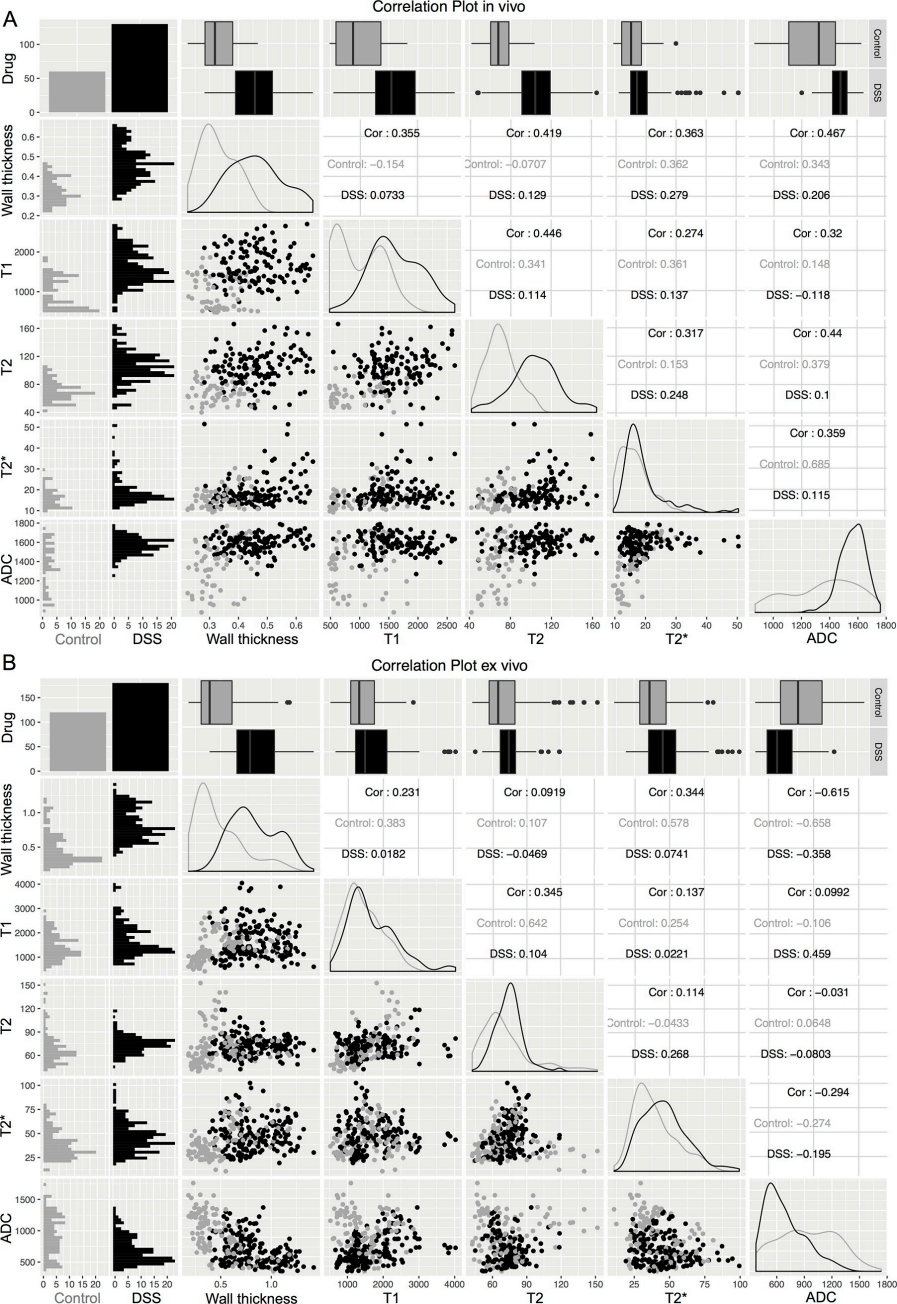
**Correlation plots for the imaging parameters acquired in *in vivo* (A) and *ex vivo* measurements (B).** Data from DSS-treated animals are displayed in black, while data from control animals are displayed in grey. Analyzed variables included wall thickness, T1-, T2- and T2*-relaxation times and the apparent diffusion coefficient (ADC). In the upper row boxplots following the Tukey definition are given to depict the distribution of the obtained parameters. Also compare Table 3A presenting p-values for the assessment of significant differences of the parameters between DSS-treated animals and controls. Frequency distribution plots along the diagonal and histograms in the left column aid to further visualize the distributions of the analyzed parameters. Dotplots below the diagonal illustrate the correlations of all possible parameter combinations, the corresponding correlation coefficients are given above the diagonal as 3 distinct r values (combined correlation Cor, controls only, DSS-treated only).

To assess correlations between the obtained parameters, correlation plots were built (Fig 5A and 5B), separated into *in vivo* (A) and *ex vivo* (B) measurements.

Highest positive correlation in the *in vivo* measurements was observed between T1- and T2-relaxation times (r = 0.446, p < 0.001). This correlation however vanished when only DSS-treated animals were analyzed (r = 0.114, p = 0.198). No significant negative correlations were observable among the *in vivo* measurements (the only negative correlation between T1-relaxation time and ADC in the DSS subgroup was weak and non-significant (r = -0.118, p = 0.181)).

In analogy to the *in vivo* measurements, *ex vivo* parameters showed significant positive correlation between T1- and T2-relaxation times (r = 0.345, p < 0.001), which also turned insignificant when excluding control animals (r = 0.104, p = 0.165). In addition, a highly significant negative correlation between wall thickness and ADC was observable in the *ex vivo* measurements (r = -0.615, p < 0.001).

Significant differences between DSS-treated and control animals were observed regarding wall thickness, T1- and T2-relaxation times and ADC (*in vivo*) and wall thickness, T2-relaxation time and ADC (*ex vivo*, compare Table 3A).

**Table 3:** Statistical comparison between DSS-treated animals and controls.

A) DSS vs. Control	*In Vivo*	*Ex Vivo*	B) *In Vivo* vs. *Ex Vivo*	DSS	Control
**Wall thickness**	0.003	*<0*.*001*	**Wall thickness**	*<0*.*001*	*0*.*21*
**T1**	*0*.*003*	*0*.*17*	**T1**	*0*.*98*	*0*.*03*
**T2**	*<0*.*001*	*0*.*04*	**T2**	*<0*.*001*	*0*.*89*
T2*	*0*.*47*	*0*.*13*	T2*	*<0*.*001*	*<0*.*001*
**ADC**	0.004	*0*.*007*	**ADC**	*<0*.*001*	*0*.*007*

(A) p-values for the comparison of the parameters wall thickness, T1 time, T2 time, T2* time and apparent diffusion coefficient (ADC) between DSS-treated animals and controls separated for *in vivo* and *ex vivo* imaging.

(B) p-values for the comparison of the same parameters between *in vivo* and *ex vivo* imaging, separated for the subgroups of DSS-treated animals and controls.

p-values in Italics were calculated using Mann-Whitney U tests due to a significant deviance from normal distribution of at least one subgroup. Remaining p-values were calculated using Student’s t-tests.

Moreover, differences between *in vivo* and *ex vivo* measurements were significant regarding wall thickness, T2- and T2*-relaxation times and ADC (DSS subgroup) and T1- and T2*-relaxation times and ADC (control subgroup, Table 3B).

### Comparison of machine learning algorithms

In an initial screening procedure, avNNet featured high sensitivity and specificity, highest overall accuracy and the highest Youden-Index (Table 4). The Blackboost algorithm and Boosted Smoothing Splines however featured slightly higher sensitivities than avNNet, but considerably lower specificities and lower overall accuracy, so that the avNNet was further evaluated in the remainder of the study.

**Table 4:** Diagnostic results of a screening procedure among different machine learning algorithms.

Algorithm	Sensitivity [%]	Specificity [%]	Accuracy [%]	Youden-Index
Model Averaged Neural Network (avNNet)	92.90	80.00	88.16	0.73
Random Forest	92.26	70.00	84.08	0.62
Support Vector Machine with Radial Kernel	92.26	67.78	83.27	0.60
Multilayer Perceptron	87.10	76.11	83.06	0.63
Blackboost	94.52	58.33	81.22	0.53
Boosted Logistic Regression	82.58	66.11	76.53	0.49
Boosted Smoothing Spline	95.48	43.89	76.53	0.39
Extreme Learning Machine	82.26	57.22	73.06	0.39

### Evaluation of the Model Averaged Neural Network

**Fig 6:**
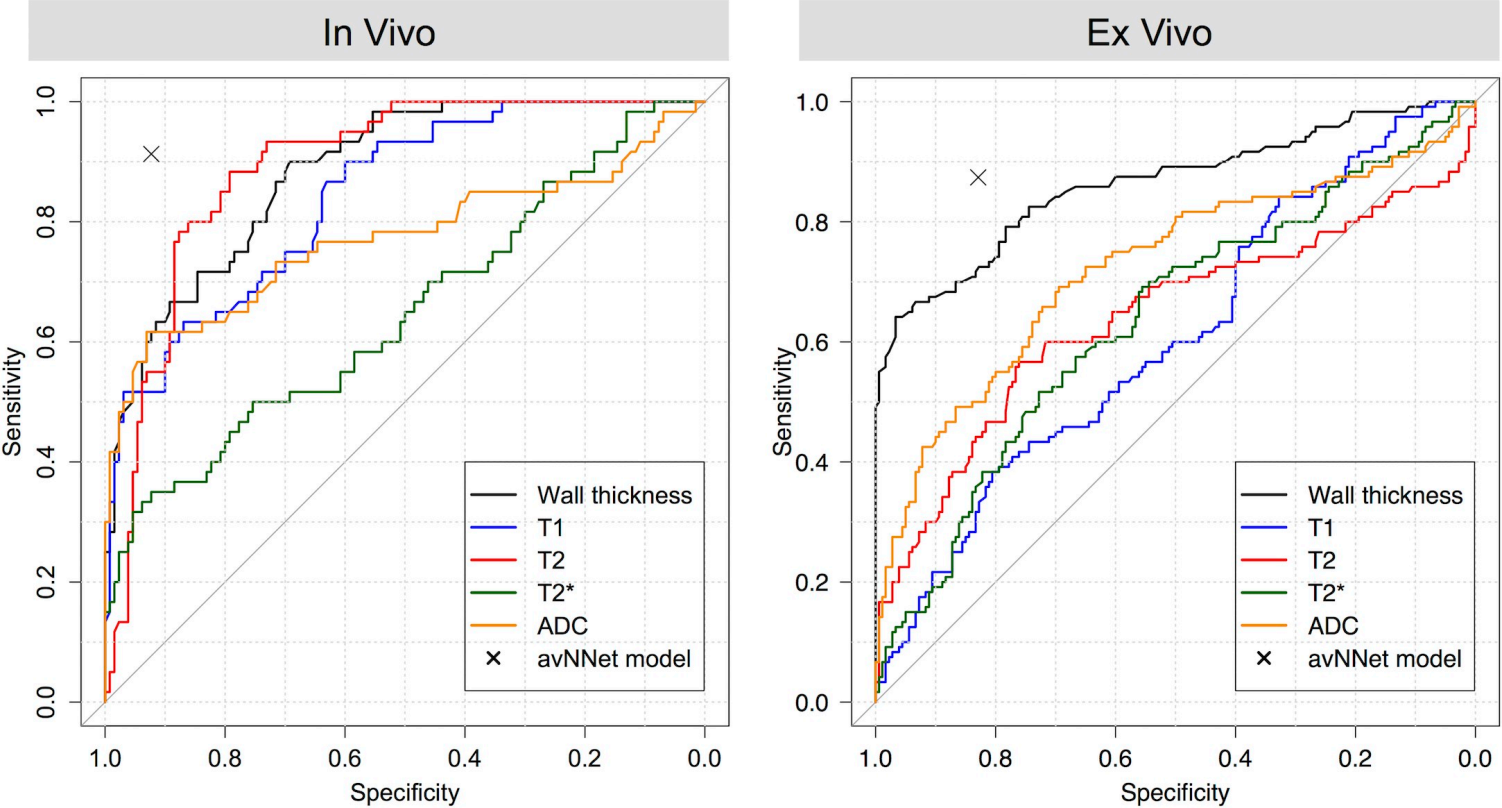
Receiver Operating Characteristic (ROC) curves for the single predictors. ROC curves are depicted for the single predictors wall thickness (black), T1- (blue), T2- (red), T2*- (green) relaxation times and ADC (orange) for *in vivo* (left) and *ex vivo* measurements (right). Optimal cutoff values were calculated via the Youden-Index and are listed in Table 5. The diagnostic values for the Model Averaged Neural Network (avNNet) are indicated with a black cross.

The resulting avNNet outperformed the predictive capacities of most single parameters (Fig 6, Table 5) and performed in no case significantly worse compared to any single value.

**Table 5:** Comparison between the predictive capacities of the acquired imaging parameters when used alone and when combined to a Model Averaged Neural Network (avNNet).

** **	**In Vivo**								
**Parameter**	**Cutoff**	**Sensitivity**	**p**	**Specificity**	**p**	**PPV**	**p**	**NPV**	**p**
**Wall thickness [mm]**	0.408	65.2%	<0.001	88.5%	0.625	93.8%	0.108	48.9%	<0.001
**T1 time [ms]**	1296	73.9%	<0.001	78.8%	0.065	90.3%	0.017	53.2%	<0.001
**T2 time [ms]**	89.238	76.1%	<0.001	90.4%	1	95.5%	0.551	58.8%	<0.001
T2* time [ms]	15.11	76.8%	0.003	55.8%	<0.001	82.2%	<0.001	47.5%	<0.001
**ADC [10**^**−6**^ **mm**^**2**^**/s]**	1444	93.4%	0.640	71.1%	0.003	89.6%	0.003	80.4%	0.949
**avNNet Model**		91.3% (86.6–96.0%)		92.3% (85.1–99.6%)		96.9% (94.0–99.9%)		80.0% (69.9–90.1%)	
** **	**Ex Vivo**								
**Parameter**	**Cutoff**	**Sensitivity**	**p**	**Specificity**	**p**	**PPV**	**p**	**NPV**	**p**
**Wall thickness [mm]**	0.629	77.6%	0.015	79.5%	0.424	85.5%	0.096	69.4%	0.008
**T1 time [ms]**	1184.9	80.8%	0.134	38.5%	<0.001	67.3%	<0.001	56.3%	<0.001
**T2 time [ms]**	66.62	73.2%	<0.001	53.0%	<0.001	70.9%	<0.001	55.9%	<0.001
T2* time [ms]	42.87	54.1%	<0.001	67.5%	<0.001	72.2%	<0.001	48.4%	<0.001
**ADC [10**^**−6**^ **mm**^**2**^**/s]**	750	69.4%	<0.001	70.1%	0.004	78.3%	<0.001	59.4%	<0.001
**avNNet Model**		87.4% (82.6–92.2%)		82.9% (76.1–89.7%)		88.9% (84.3–93.5%)		80.8% (73.8–87.9%)	

Cutoff values for the single parameters were determined using the Youden-Index from the ROC curves in Fig 5. Sensitivities, specificities, positive predictive values (PPV) and negative predictive values (NPV) for the distinct cutoff values are given in percent. In addition, sensitivities, specificities, PPV and NPV of the Model Averaged Neural Network (avNNet) are listed, with the 95% confidence intervals given in brackets. The p-values derive from a comparison of the accuracy measures of the single parameters at their respective cutoffs with the values of the avNNet using exact binomial tests.

In the *in vivo* analysis, T2 time featured the ROC curve closest to the top-left edge (Fig 6), resulting in a sensitivity of 76.1%, specificity of 90.4%, PPV of 95.5%, and NPV of 58.8% when using the optimal cutoff of 89.238 ms (Table 5). T2 time as a single predictor was however significantly outperformed in terms of sensitivity and NPV by the avNNet with its accuracy measures of 91.3% sensitivity (p < 0.001), 92.3% specificity (no significance), 96.9% PPV (no significance) and 80.0% NPV (p < 0.001, also compare Table 5). Regarding the *in vivo* analysis, the avNNet was slightly but not significantly outperformed by the ADC value as a single predictor in terms of sensitivity and NPV (93.4% vs. 91.3% and 80.4% vs. 80.0%, respectively), but featured significantly higher specificity and PPV values (92.3% vs. 71.1%, p = 0.003 and 96.9% vs. 89.6%, p = 0.003, respectively).

In the *ex vivo* analysis, wall thickness as the best performing single parameter (sensitivity 77.6%, specificity 79.5%, PPV 85.5%, NPV 69.4%) was outperformed by the avNNet in terms of sensitivity and NPV (avNNet sensitivity 87.4%, p = 0.015; specificity 82.9%, no significance; PPV 88.9%, no significance; NPV 80.8%, p = 0.008. Also compare Table 5). The avNNet was not outperformed by any single parameter in the *ex vivo* analysis.

A visual impression of the avNNet’s performance in comparison to all single parameters is given in Fig 6, the detailed values and results of the statistical tests are listed in Table 5.

Overall diagnostic accuracy (percent classified correctly) for the avNNet was 88.0% (95% CI: 84.7–90.7%). When defining the presence of colitis not by DSS-treatment but rather by disease specific scores ≥1, diagnostic accuracy was comparable (88.1%; 95% CI 84.8–90.9%). When using a histology score of ≥ 2 as a criterion for colitis definition, diagnostic accuracy was slightly reduced (85.0%; 95% CI 84.7–90.7%).

### Final model development and preparation of an online tool for colitis assessment in mice

The final model (trained on the complete dataset) was compiled to a web application publically accessible via https://stoevne.shinyapps.io/MouseColitis/.

## Discussion

The sensitive and quantitative analysis of structural changes of mouse colon tissues associated with experimentally induced IBD is urgently required for an objective evaluation of disease progression. However, evaluation of IBD in mice remains a challenging task due to the small structures involved in inflamed bowels. Analysis is moreover complicated by bowel movements during *in vivo* examinations that can only be partly suppressed by butylscopolamine application.

This study presents *in vivo* and *ex vivo* protocols to predict the presence of DSS-induced murine colitis using a machine learning algorithm that combines multiple parameters. Several studies have been published investigating MR imaging of IBD in mice [15–21]. These studies focused on single [16,17,20] or only few parameters [15,18,21] without combining them in a holistic approach. Wall thickness as a useful predictive parameter for the presence of colitis was confirmed in several studies [15–18,21]. In addition, T2w signal intensity could be shown to be a parameter related to disease activity [15,21], which is in accordance with our results (see Table 3A). An extensive MR imaging analysis was performed by Mustafi et al. [18], investigating T1- and T2-relaxation times in a mapping approach, wall thickness and dynamic contrast enhancement. Mustafi et al. also described increased T2-relaxation times for colitis, which was confirmed by our results (Table 3A). In contrast to our study, no significant differences between T1w values of inflamed and control colon were reported by these investigators [18].

In addition to already published studies, this study provides several aspects not covered by previous works: We provide a combined approach applicable for *in vivo* as well as *ex vivo* imaging, with an investigation of several parameters and their predictive capacities alone and in combination. The resulting model was cross validated to ensure generalizability and was made publicly available to be used by other researchers. However, machine learning algorithms largely function as “black box”, and it remains unclear which features affect the final result to which specific extent.

Concerning the single parameters within the presented study, a significant increase of wall thickness in colitis both *in* and *ex vivo*, an increase of T1-relaxation time *in vivo* and changes of diffusion capacity *in vivo* and *ex vivo* were observed. T2-relaxation times were increased in DSS-treated animals compared to sham-treated animals in both *in vivo* and *ex vivo* analyses. T2*-relaxation times between DSS- and sham-treated animals did not differ significantly, neither in *in vivo* nor *ex vivo* imaging. A striking finding was the increase of colon wall diffusion capacity in mice suffering from colitis in *in vivo* imaging. This has formerly been described for necrotizing enterocolitis in rodents [29] along with increased T2-relaxation times, pointing to a possible necrotizing component in DSS-induced colitis in mice. In contradiction to these *in vivo* findings, inflamed colon walls showed decreased ADC values in *ex vivo* imaging, which is in line with clinical studies in human patients describing a significant decrease of ADC values from normal colorectal tissue to healing lesions to active UC [30]. However, quantitative ADC measurements have also been described to feature poor discriminatory ability for segmental disease activity [31].

Overall, wall thickness, T2*-relaxation time and ADC were determined significantly different between *in* and *ex vivo* analyses, T1-relaxation time differed significantly between *in* and *ex vivo* in the control group and T2-relaxation time in the DSS group (Table 3B).

These results altogether suggest components influencing the accuracy of the measurements, probably at least in part attributable to bowel movements and partial volume effects especially in the *in vivo* subgroup, or altered relaxation times and diffusion coefficients due to preparation and embedding of the colon, or due to the long-lasting overnight imaging (approx. 7.5 h) causing tissue alterations over time. The presented model however is able to distinguish between DSS-treated and control animals with high accuracy (Table 4). In this regard, diagnostic accuracy was even higher for *in vivo* than for *ex vivo* imaging. A reason to explain this might be that wall thickness as the most useful predictive parameter in the *ex vivo* analysis plays a more important role when bowel movements are absent, and is less reliable when measured in structures affected by peristaltic waves, which is not avoidable in the *in vivo* situation. Most probably, this disadvantage is more than compensated by relaxation times and diffusion capacity that can be determined more accurately in the *in vivo* situation under conditions of intact blood perfusion. This fact has been described in former comparisons between *in vivo* and *ex vivo* data with significant differences between relaxation times of live tissue and fresh tissue samples [32], as well as dependencies on tissue temperature [33]. In particular, the observed significant differences of T2* relaxation times between *in* and *ex vivo* measurements are not surprising, as T2* relaxation times depend on a variety of physiologic features including the ratio of deoxyhemoglobin to oxyhemoglobin in the blood, blood volume and blood flow [34,35]–parameters altered by nature when performing *ex vivo* analyses. Additional tissue changes due to the embedding in agarose and the accompanying sudden temperature changes might also influence relaxation times and lead to more reliable measurements in the *in vivo* situation.

We are aware that our study has several limitations: The group of DSS-treated animals has to be considered heterogeneous with disease specific scores ranging from 1 to 13.5 (Fig 2A) and histology scores ranging from 1–6 (Fig 2B), possibly at least in part attributable to a known substantial variability in different lots of this substance [10]. In this pilot study, the heterogeneous group of DSS-treated animals was subsumed, and DSS treatment served as a reference condition chosen for mainly two reasons: 1) the model aimed to predict a dichotomous outcome, so that choosing the dichotomous variable of DSS-treatment seemed a reasonable approach, and 2) a definite gold-standard for IBD evaluation in mice remains to be established, as a plethora of different scoring systems exists [36]. Most scoring systems involve semi-quantitative or even subjective criteria, do not correlate perfectly with each other and offer no clear cutoffs for the presence of significant colitis. To add to these concerns, our study determined an only moderate correlation coefficient between clinical scoring and histology of 0.455 (Fig 3). Though Walldorf et al. calculated a slightly higher correlation coefficient of 0.621 [20], discrepancies similar to those of our study have also been described in humans: In human patients, histology scores were also moderately correlated to endoscopy scores, but especially mild disease activity in endoscopic scoring was distributed over the entire range of histologic grades [37]. The same tendency became apparent in this study’s correlation analysis, with the most imperfect correlations observed in mice with disease specific scores ≤ 4, but histology scores scattered over the entire spectrum (Fig 3). Given the fact that several animals of our study featured high scores in histology but low scores in disease specific scoring, we felt that the most sensible, objective reference condition was DSS-treatment. When however choosing reasonable cutoffs for disease specific scores and histology scores, the model nonetheless performed accurate as well. We however did not aim to question or redefine different evaluation standards established by different groups, but to prove that a combination of multiple image parameters can increase diagnostic accuracy. The choice of a particular reference condition–though of clinical importance–should thus be regarded secondary in this context.

Correlations of image parameters with measures of disease activity have already been performed. Melgar et al. for example calculated correlations of colon wall thickness and T2w signal with a clinical scoring system and other parameters of disease activity, and speculated on the advantages of combining different parameters to a model [21]. We are well aware that the results of this pilot study with a model predicting a binary outcome represents just a first step on the long path of comprehensive IBD activity assessment. In future studies needing higher sample numbers, the model could be further improved to not only predict the dichotomous outcome of the presence of colonic inflammation, but to directly calculate MRI-derived colitis scores to quantify disease activity. This would of course require a valid gold standard, but have direct implications on diagnosis and offer results transferable to clinical questions as a non-invasive substitution for colonoscopic examinations. Though a quantitative MRI-based colitis evaluation will probably not be appropriate for high-thoughput screenings due to the need for animal preparation before starting the imaging protocol, it still offers a less biased technique to grade disease activity in contrast to endoscopic scoring, which largely depends on the experimentator’s experience in colonoscopy.

The model of this study was validated with a modified Leave-one-out cross-validation, which is appropriate considering the relatively small sample number. With the animal numbers used for this study it was not possible to initially exclude a larger subset of data for testing while simultaneously being able to calculate reliable accuracy measures. It is common practice to then use resampling methods such as cross validation to estimate the generalizability of a model as done in this study [38,39].

We did not use dynamic contrast enhancement (DCE) and total contrast enhancement of colonic walls which have been described previously as parameters allowing visualization of inflammatory activity [15,18]. However, as contrast media application can only be performed during *in vivo* imaging, we did not include contrast-enhanced sequences in order to maintain comparison between the *in* and *ex vivo* subgroups. In future studies, the inclusion of DCE may further increase diagnostic accuracy in the *in vivo* subgroup.

As an outlook, the present work serves as a pilot-study with the future aim to develop an MR-derived colitis score. Such a score would allow more elaborate non-invasive, unbiased diagnosis including longitudinal assessments e.g. under novel therapeutic agents. Up to now, it however remains unclear whether a quantitative model will be able to sufficiently judge on disease severity or progression. As a proof-of-concept, the presented online tool allows researchers from external groups to use this once created model for assessment and evaluation of their own measurements, without having to re-establish a model on their own. The presented tool is self-explanatory to use and requires no programming skills. Further extensions of this tool in future studies are planned.

To conclude, the advantages of combining multiparametric imaging with machine learning algorithms in a holistic approach is expandable to several other clinical and preclinical questions including inflammation, infection and oncology, and will lead to increased diagnostic accuracy.

## Supporting information

S1 Fig Results of the principal components analysis. The principle components are depicted on the axis of abscissas, and their cumulative proportion of variance explained on the axis of ordinates.
(TIFF)
