# Supplementary figures and images for: Application of machine learning algorithms for multiparametric MRI-based evaluation of murine colitis
Source: PLoS One. 2018 Oct 26;13(10):e0206576. doi: 10.1371/journal.pone.0206576 (PMC6203400; doi:10.1371/journal.pone.0206576)

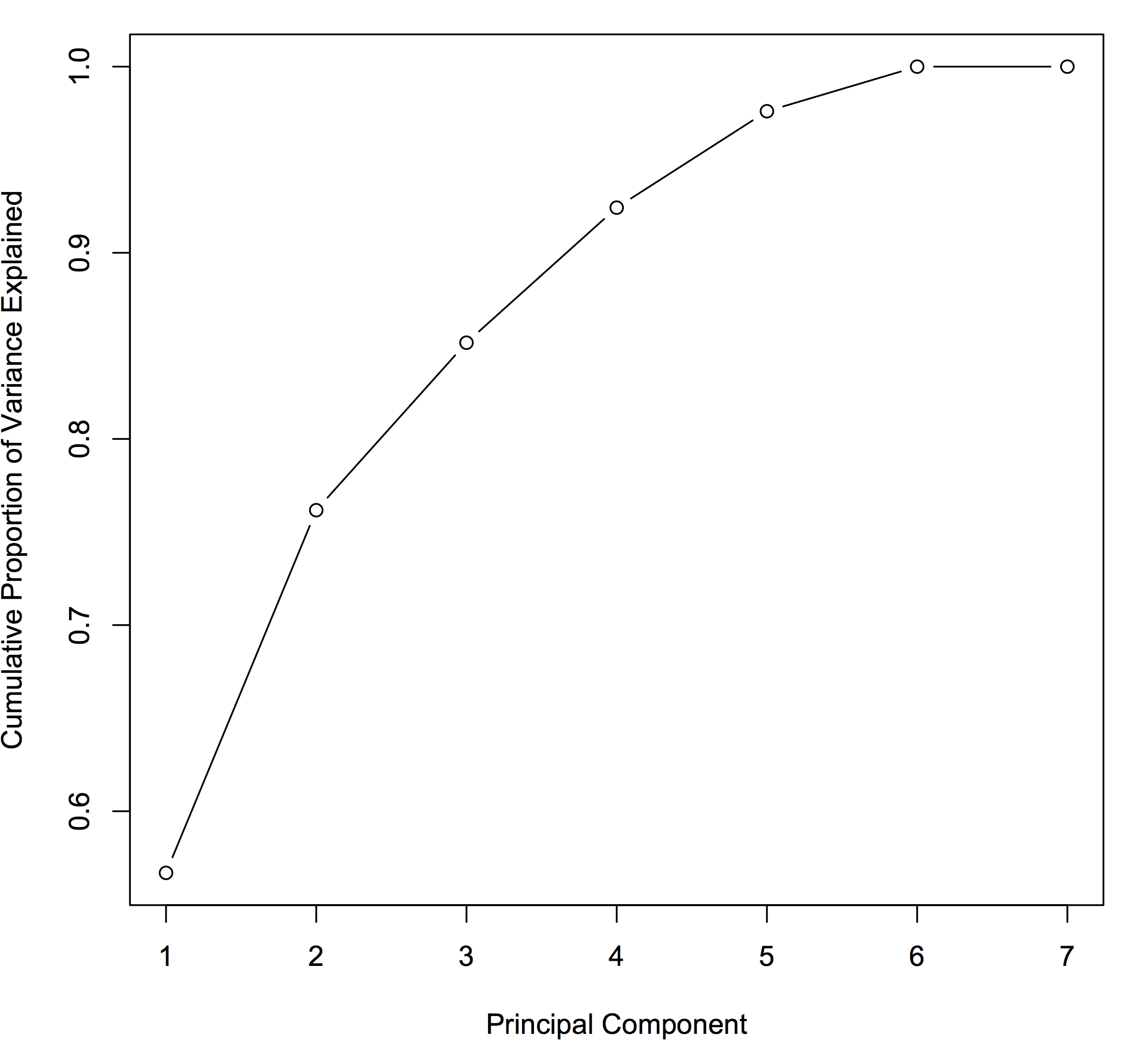

Supplement: S1 Fig — The principle components are depicted on the axis of abscissas, and their cumulative proportion of variance explained on the axis of ordinates. (TIFF) [file pone.0206576.s001.tiff]
